# Lateral heat flux reduction using a lock-in thermography compensation method

**DOI:** 10.1038/s41598-023-44128-0

**Published:** 2023-10-10

**Authors:** Johannes Rittmann, Marc Kreutzbruck

**Affiliations:** Institute for Plastics Technology, University of Stuttgart, Pfaffenwaldring 32, 70569 Stuttgart, Germany

**Keywords:** Mechanical engineering, Mid-infrared photonics, Characterization and analytical techniques

## Abstract

The naturally diffusive heat flow in solids often results in differences in surface temperatures. Active thermography (AT) exploits such differences to gain information on the internal structure, morphology, or geometry of technical components or biological specimens. In contrast to sound or light waves, thermal waves are lossy; consequently, it is difficult to interpret measured 2D temperature fields. Most AT evaluation methods are based on 1D approaches, and measured 3D heat fluxes are frequently not considered, which is why edges, small features, or gradients are often blurred. Herein, we present a method for reducing the local temperature gradients at feature areas and minimizing the induced lateral heat flux in optical lock-in thermography (LT) measurements through spatial- and temporal-structured heating. The vanishing lateral gradients convert the problem into a 1D problem, which can be adequately solved by the LT approach. The proposed compensation method can bypass the blind frequency of LT and make the inspection largely independent of the excitation frequency. Furthermore, the edge sharpness and separability of features are improved, ultimately improving the feature-detection efficiency.

## Fundamentals of signal formation and structured heating

The paper is divided into three sections. The first part explains the basics of the lock-in thermography compensation method (LTC). In the second part, the numerical limitations of the method are described regarding the blind frequency (BF) shift, feature detection, depth range, feature separation, and lateral resolution. In the third part, the numerical results are validated using experimental data.

For a temperature gradient, $\nabla T$, heat flows from the warm location to the cold location according to Fourier’s law, $\dot{\vec{q}} = - \lambda \nabla T$. The thermal conductivity ($\lambda$) indicates the relationship between the temperature gradient ($\nabla T$) and the induced heat flux density ($\dot{\vec{q}}$). For the 1D case (e.g., a bar or a homogeneous large plate without features), analytical equations can be used to describe the propagation of thermal waves in solids. Under these assumptions, the effects of the layer thicknesses or material inhomogeneities on the amplitude and phase in LT can be elucidated^1^. Notably, the present-day LT was developed based on the mathematical description of the photothermal effect provided in several foundational studies^2–4^. LT is an inspection method in AT, wherein a (harmonically) modulated excitation source is used to heat the surface temperature of a specimen^5, 6^. To analyze the response signals, LT evaluation is performed for each pixel of the infrared (IR) camera, conventionally by a pixel-wise Fourier transform, which functions like a narrow bandpass filter that only considers signals of the frequency of (harmonic) excitation. LT provides two essential pieces of information: the phase and amplitude. The phase compares the delay between the excitation and measurement signals, whereas the amplitude describes the pixel-wise temperature oscillation of the thermal wave in the measurement signal.

To date, AT has been used mainly for testing materials, electronic components, and semiconductors^6^. However, research exploring new application areas is ongoing. Generally, thermography plays a minor role in medicine^7–9^. Previous investigations, although predominantly numerical, have shown that cancer can be detected by modulating the excitation frequency in AT^10^. Early onset, near-surface melanoma can be detected with LT at excitation frequencies of 100 mHz; however, deep and critical melanoma require low excitation frequencies, which result in blurred images^11^. The minimization of lateral heat flux by active cooling improves the resolution^12^.

Additionally, numerous AT measurement and evaluation methods exist, such as thermal wave imaging^13^ and thermal wave radar measurements^14, 15^. However, these methods, including the LT, are based on the pixel-wise evaluation of the measured thermal wave and do not consider the lateral heat flux, inevitably resulting in the blurring of inhomogeneous areas. In an iterative numerical approach based on the Levenberg–Marquardt algorithm, hidden structures were constructed from thermograms after flash excitation, partially considering the lateral heat fluxes^16^. The virtual-wave concept^17^ attempts to consider the lateral heat flux by numerically solving the inverse problem of heat conduction. The concept explores the link between the point heat source in a diffusion-free space and the diffusion-affected reality.

The lateral resolution, $\delta_{Abbe}$ (1), at the surface for a heat-generated delta source at depth *d* based on the equation for the optical Abbe diffraction is described in^18^ and depends not on the thermal properties of the specimen, but on the geometrical properties and the signal-to-noise ratio (SNR).

1 $$\delta_{Abbe} = 2\pi \frac{d}{{\ln \left( {SNR} \right)}},$$

Structured heating is another method for accounting for lateral heat flow, which was first implemented in^19^. A laser beam was split into two coherent beams, and their interference in a specimen was exploited to obtain the depth information of features.

In^20^, a liquid crystal display projector served as an optical heat source for AT pixel-wise structured heating. However, due to the small amount of thermal radiation, the surface could only be heated marginally. For LT measurement, the pixel-wise phase difference, amplitude difference, and average temperature deviation are compensated. The objective is to obtain a phase and amplitude image that is highly homogeneous in a LT measurement. The compensation is completed when no feature information is observed. The lateral resolution is improved by compensation using a factor of ~ 2^20^. Notably, the described method compensated for only near-surface lateral-heat-conduction effects.

In^21^, a digital micromirror device (DMD) chip was combined with a near-infrared (NIR) laser to achieve a high-power, structured light source (20 W input power, 4.4 W optical output power). In the centerline of two parallel and 180° temporally phase-shifted lines of the same frequency, the alternating thermal field could be canceled by destructive interference. By observing the centerline, disturbances of a homogeneous material could be detected. In^22^, a DMD chip and a 30 W NIR laser were used to visualize features. The simultaneous projection of two thermal patterns of different excitation frequencies (LT excitation at 2 Hz and 2.2 Hz) could be spectrally filtered from the thermogram.

In^23^, a DMD projector (optical output power = 85 W) was used to perform thermal super-resolution measurements through 2D projected patterns on metal specimens, enabling precise feature detection. However, this method was time-consuming.

Next, an LTC method based on the approach employed in^20^, which selectively influences the lateral heat flux in a LT measurement, is presented.

### Fundamental operating principle and method description of LTC

In the 3D case, lateral heat flux inevitably occurs after a local temperature change, probably due to nonuniform heating or inhomogeneous heat conduction. LT measures the local phase shift and the local amplitude of a measuring point in relation to the excitation signal. At inhomogeneous areas, the measurement signal changes, with the local amplitude increasing for inhomogeneities with low heat capacities (such as an air inclusion). The phase difference is not unique, and no trivial statement can be made about its value or sign^24^. The phase behaves differently depending on the feature geometry, heat-transfer coefficient, depth location, and excitation frequency. It is strongly influenced by the local lateral heat flux and can vary within a constant feature depth.

LTC requires an illumination source (maximum illumination intensity: *I*_max_) with temporal and spatial adaptability. With this, the locally variable excitation can be exploited to minimize the mean lateral heat flux within the LT measurement period. If an excitation scenario is found for which the lateral heat flux is minimized, lower temperature gradients will occur within the specimen.

The following procedure shows a possible strategy for an iterative excitation scenario that decreases the lateral heat flux in the specimen, starting from a LT measurement. The (local) sinusoidal excitation (I(*x*,*y*,*t*)) is divided into three interdependent excitation and measurement parameters, whereas the general excitation structure of the LT is maintained:

2 $$I\left( {x,y,t} \right) = \frac{{I_{max} }}{2}*Off\left( {x,y} \right) + \frac{{I_{max} }}{2}*Amp\left( {x,y} \right)*\sin \left( {wt + \frac{2\pi }{{360}}Phi\left( {x,y} \right)} \right),$$

*I*_max_, *Phi*(*x*,*y*), *Amp*(*x*,*y*), and *Off*(*x*,*y*) describe the maximum excitation intensity, local phase shift of the excitation and measurement signals, local amplitude of the excitation and measurement signals, and local DC heating component of the excitation in the form of the average local temperature increase within one period in the specimen due to the excitation, respectively. The measured amplitude of a periodic signal reflects the periodic temperature variation occurring within the measurement period. Areas with higher amplitudes correspond to greater temperature variations, resulting in a temperature gradient toward local regions with lower amplitudes. The behavior of the offset follows a similar pattern. When two signals are temporally shifted relative to each other (∆*Phi*_meas_ ≠ 0), a lateral temperature gradient is established at every point as function of time (except at the intersections of the functions). By homogenizing the amplitude, phase, and offset through an iterative process, the lateral temperature gradients are reduced, thereby aligning the thermal problem closer to a 1D model along the depth-direction, where all lateral heatflow disappears.

Other excitation approaches can also minimize the local lateral heat flux. For example, locally resolved pulse excitation with pixel-wise variable starting times, illumination durations, and intensities could be used. This excitation approach is efficient, and the LTC results can be determined from the LT results and the inhomogeneous excitation by a simple calculation step.

The LTC method focuses on iteratively minimizing the lateral heat flux in the specimen by the specific local adjustment of the three excitation parameters. If an excitation approach exists for which the measured signal provides a homogeneous response signal (*Phi*_meas_, *Amp*_meas_, and *Off*_meas_), perfect compensation would be achieved. A homogeneous response signal is caused by an in-lateral-direction homogeneous temperature field in the specimen. Notably, perfect compensation in nonhomogeneous structures is only possible if *Amp*_ex_ = *Off*_ex_ = 0 (trivial solution). In this scenario, no feature information is acquired. In the nonhomogeneous case, a local temperature gradient inevitably occurs, due to an inhomogeneous excitation or the inhomogeneity of the structure, inducing a lateral heat flux.

If a nontrivial excitation approach exists for which the response signal of the LT measurement is highly homogeneous, the LTC results can be directly derived from the parameters of the employed excitation scenario. Since no lateral diffusion processes occur in the excitation source, the features in the specimen can be displayed more sharply than in LT or other point-based evaluation methods. The LTC results are transferred to the virtual plane of the excitation.

**Figure 1:**
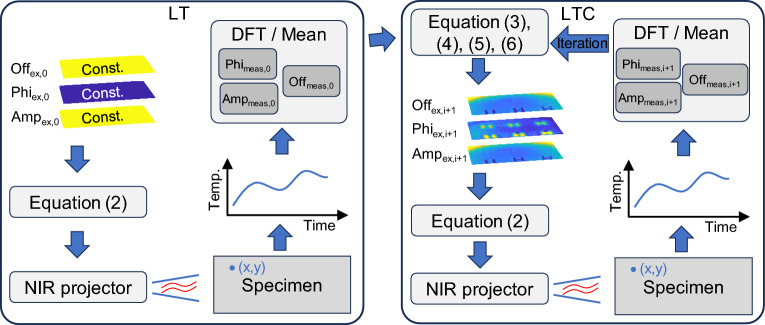
Flowchart of the LTC.

As there is no a priori knowledge of the desired feature geometry, the first iteration of the LTC starts with LT. Within Eq. (2), the phase is assumed to be constant (*Phi*_ex_ = 0) and the amplitude and offset are set to *Amp*_ex_ = *Off*_ex_ = 1. *I*_max_ is selected according to the test requirements or the excitation-source limitations to avoid thermal damage to the specimen. Alternatively, it is set according to the maximum intensity of the excitation source. The LT affords the values of *Phi*_meas_, *Amp*_meas_, and *Off*_meas_ for iteration 0 of the LTC. Based on the results, the local excitation parameters for the subsequent iteration are determined. The excitation phase (*Phi*_ex, i + 1_) is trivially compensated, and the respective differences between the previous excitation and the previous result are considered for the subsequent iteration. For the amplitude and offset, the subsequent excitation amplitude (*Amp*_ex, *i* + 1_) and the excitation offset (*Off*_ex, *i* + 1_) are decreased by a factor such that the local amplitude and the offset are larger than the smallest amplitude and offset in the result. This process decreases the amplitude and the offset with every iteration. Thus, the optimization-loop limit converges to *Amp*_ex_ = *Off*_ex_ = 0. Thus, *Phi*_ex_ becomes indeterminate. The phase and amplitude are iterated as described in^20^ and are given in Eqs. (3) and (4). A modified iteration is used for the excitation offset. Since the offset must always be larger than or equal to the amplitude, the local excitation offset is calculated as the maximum value of the excitation offset and the excitation amplitude, as shown in Eqs. (5) and (6). Numerically, the LTC can be implemented robustly. In experiments, inhomogeneous illuminations of the excitation source hamper the iteration procedure and, therefore, require an additional stability criterion. Instead of employing the minimum amplitude and offset as reference for iteration, empirical evidence has demonstrated that using the 20% percentile values for amplitude and offset yields a more robust approach. Ranges below the 20% percentile limit are not compensated in the amplitude or offset parameter. Although this causes the results to converge more slowly, the results can be mapped more robustly unlike in the case with the iteration rules described in^20^. The stability criterion has a disproportionate influence on noise-retained structures, but it has no impact on features with signal levels above the 20% percentile. Within one iteration, the noise of the excitation (previous measurement) overlaps with the noise of the current measurement, making the noise in the LTC result larger than the LT noise. Random noise, however, is compensated by the iteration rule over several iterations, whereas systematic noise increases with increasing iterations. In the experiment, the excitation was, therefore, smoothed using a moving average filter with a kernel size of approximately 1 mm × 1 mm. Since the filtering only occurs on the excitation side, the result is still overlaid by the thermal noise of the LT. A flowchart of the LTC is shown in Fig. 1.

3 $$Phi_{{ex,{ }i + 1}} = Phi_{{ex,{ }i}} - Phi_{{meas,{ }i}} ,$$

4 $$Amp_{{ex,{ }i + 1}} = Amp_{{ex,{ }i}} \cdot\frac{{\min \left( {Amp_{{meas,{ }i}} } \right)}}{{Amp_{{meas,{ }i}} }},$$

5 $$Off^{*}_{{ex,{ }i + 1}} = Off_{{ex,{ }i}} \cdot\frac{{\min \left( {Off_{{meas,{ }i}} } \right)}}{{Off_{{meas,{ }i}} }},$$

6 $$Off_{{ex,{ }i + 1}} = \max \left( {Amp_{{ex,{ }i + 1}} ,{ }Off^{*}_{{ex,{ }i + 1}} } \right).$$

### Description and implementation of the numerical LCT

The heat flux simulation is implemented using the COMSOL Multiphysics finite element software (COMSOL AB, Stockholm, Sweden). For this purpose, a polyoxymethylene (POM)-specimen with a size of 40 mm × 40 mm × 4 mm, density ($\rho$) of 1410 kg/m^3^, specific heat capacity (c) of 1500 J/(kg K), and thermal conductivity ($\lambda$) of 0.31 W/(m K) was modeled and provided with different internal geometries. The heating was applied over the top surface of the homogeneous specimen and excitation was achieved using a locally varying sinusoidal heat source according to Eq. (2). The discrete pixel size of every individual spot of the heating source was set to 100 µm × 100 µm. The same pixel size was used for numerical evaluation. The *I*_max_ of the illumination source was 800 W/m^2^. The inclusions and the backwall were considered as thermal insulators, and the lateral surfaces were modeled as an adiabatic boundary condition. A convective heat flux of 5 W/(m^2^ K) was assumed on the top surface of the specimen. The mesh was composed of a tetrahedral mesh of size 0.8 mm (mesh refinement on thin structures was set to 0.33 mm) and quadratic serendipity elements. To achieve good convergence of the numerical simulation, the mesh size was reduced by 25% for frequencies above 100 mHz. LT simulations were performed at different excitation frequencies for two periods. Per period, at 20 equally distributed time steps, the surface temperature was evaluated and superimposed with a uniformly distributed thermal noise of 20 mK. From the simulated (or later in the study measured) temperatures, the local phase and amplitude information were calculated directly using a discrete Fourier transform. The offset was calculated as the local mean temperature increase in the last period. A detailed description of the Fourier transform employed can be found in^25^. The resulting excitation phase of the LTC arises from the measured amplitude, offset, and phase values, which are merged to a new value with increased information density. Next, only the phase is described and compared, as is typical for LT evaluation.

**Figure 2:**
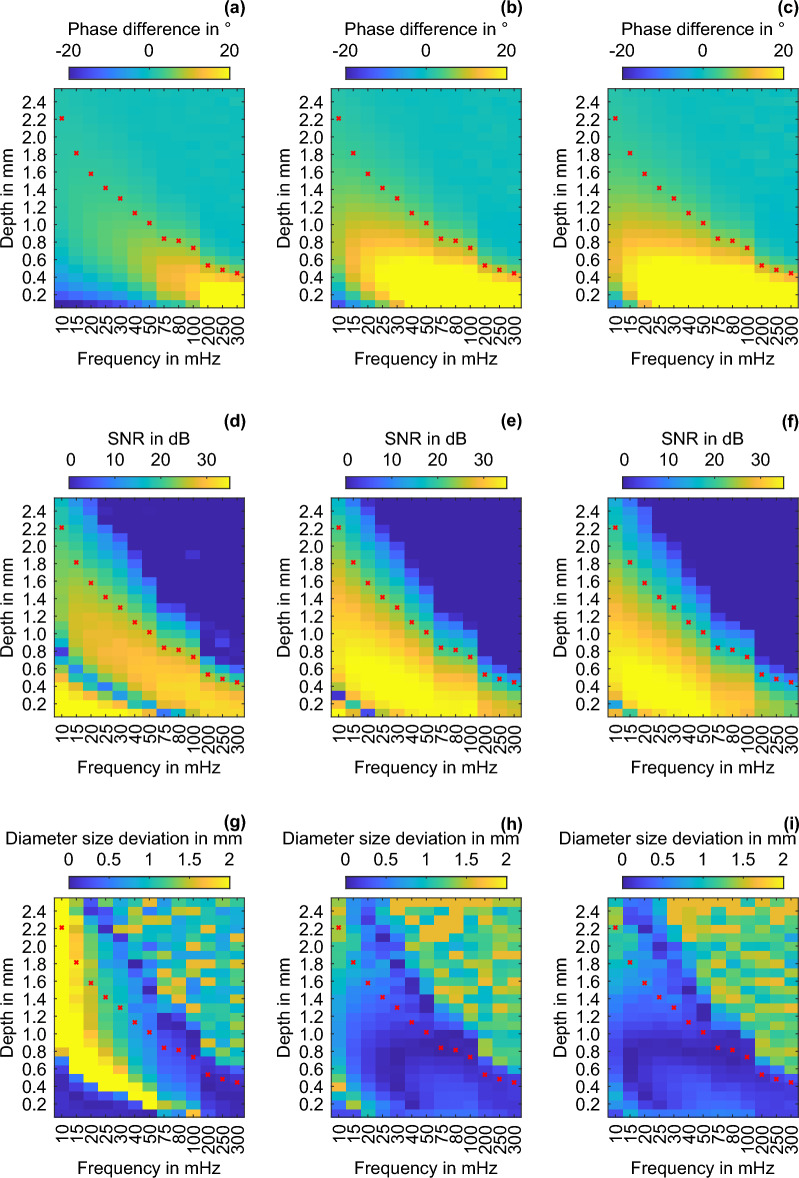
Frequency–depth images of FBH 2 for the phase difference: (**a**) LT, (**b**) LTC after six iterations, and (**c**) LTC after ten iterations; for the SNR-value: (**d**) LT, (**e**) LTC iteration six, and (**f**) LTC iteration ten; and for the diameter-size deviation: (**g**) LT, (**h**) LTC iteration six, and (**i**) LTC iteration ten. LT phase differences are displayed physically negated.

As described in^24–30^, the phase does not behave trivially, and under certain constellations, the feature-signal sign changes in relation to the background signal, making it difficult to detect features in certain depths. This zero crossing is also called BF or blind frequency–depth (BFD). For frequency and depth ranges of 10–300 mHz and 0.1–2.5 mm, respectively, three different evaluations for a 2 mm flat-bottom hole (FBH 2) in the described 4 mm thick POM specimen are shown in Fig. 2. The left column represents the classical LT; the middle, LTC after six iterations; and the right, LTC after ten iterations. For enhanced comparability with the LTC result, the LT phase difference has been negated. All subsequent statements regarding the sign of the phase difference of the LT must be considered inverted for physical correctness. The LTC phase is described in the physically correct sign. The thermal penetration depths matching the respective excitation frequency are plotted in red. With increasing frequency, the angular frequency ($\omega$) increases and the thermal penetration depth (*µ*) decreases:

7 $$\mu = \sqrt {\frac{2 \cdot \lambda }{{\rho \cdot c \cdot \omega }}} ,$$

where ${\uplambda }$, ${\uprho }$, and *c* are the thermal conductivity, density, and specific heat capacity, respectively. The upper row in Fig. 2 shows the mean phase differences of FBH 2 relative to the sound background. The middle row shows the SNR (in dB) of FBH 2 relative to the sound background. The bottom row shows the mean diameter-size deviations (in mm), based on the full width at half maximum method (FWHM) relative to the actual size of FBH 2. The BF^24, 26–30^ is a well-known problem in LT. The only solution is to use a different method, e.g., frequency-modulated thermal wave imaging^30^, or to cover the depth range of the LT with several excitation frequencies. The influence of the BF in LT in the frequency and depth space is described by the graphs in Fig. 2a,d. For low excitation frequencies and near-surface features, the phase difference is negative. As the depth or excitation frequency increases, the phase difference initially tends to zero (BFD) and subsequently increases after the sign change until it tends toward zero again at the range limit. If the magnitude of the phase difference decreases relative to the background, the feature can no longer be detected. The BFD is highlighted by vanishing SNRs surrounded by high SNRs. Above the BFD, features can be found in the LT at a high SNR. Furthermore, only a small size deviation from the actual feature size occurs (Fig. 2g). In the BFD range, the size deviation reaches the maximum because of the disappearing phase difference magnitudes. For deeper features, the SNR increases again, although the size deviation remains high and decreases only gradually due to the increasing lateral heat flux. To detect the features while preserving geometry, the LT should be performed above the BFD. The inspection range of the phase until the point where the SNR tends toward one for numerical LT is approximately 1.7 times the thermal penetration depth, consistent with the literature^27^.

The LTC solves the BF problem and extends the LT through several approaches. Through compensation, the measured amplitude and offset at inhomogeneities decrease, decreasing the lateral heat fluxes and the thermal disturbance around the features. The phase compensation homogenizes the temporal heat distribution. If the correct sign is already present in the phase image at the inhomogeneity (feature deeper than the BFD), the compensation can be stopped after only a few iterations. If, conversely, a negative phase difference is present, e.g., due to the existence of a near-surface feature at low excitation frequencies, more iterations are required until the LTC converges. In this case, the phase is influenced such that the sign of the negative phase differences changes with increasing iteration and subsequently tends toward a value characteristic of the feature size and the feature depth. In Fig. 2h,i (LTC after six and ten iterations, respectively, compared with those for the LT (g)), the size deviation of the features decreases significantly and only occurs (deviations > 1 mm) in areas where the compensation is uncompleted (Fig. 2h). The uncompleted compensation is illustrated by the sign change in phase difference images (b) and (c), as well as the area with disappearing SNRs in (e) and (f). If near-surface structures are to be examined at low frequencies, many iterations are needed. The BFD shifts to the near-surface region with increasing iteration or frequencies. If iteration 0 (LT) is evaluated together with LTC, both the depths up to the BFD can be covered by LT and the depths from the BFD can be covered by LTC with high accuracy.

In the SNR images, the LTC provides an increased SNR up to the thermal penetration depth. Beyond the thermal penetration depth, the LT has a higher SNR but also a larger size deviation. This is because, in addition to the phase, the amplitude and offset are considered for the compensation. Thus, the LTC range is approximately equal to the LT amplitude range, corresponding to the thermal penetration depth. The increased noise (excitation + measurement) induces a decrease in the SNR outside the amplitude range, although features up to ~ 1.5 times the thermal penetration depth can be observed (Fig. 2e,f). In line with the increased phase difference and sharp feature imaging, as described in^20^, the gradient at feature edges also increases. The LTC works only for defects with detectable SNR in LT. Enhancing the visibility of defects that are barely discernible lies beyond the capabilities of LTC. If a defect remains undetected, it is either due to its small size, which falls below the LT detection threshold, or its depth, which requires a lower excitation frequency for detection. By employing finite elements^16, 31, 32^, optimization techniques such as the Hamilton-Jaboci formalism^33^ or Levenberg–Marquardt algorithm^16, 34^ as well as convolution-based methods^35–40^, it becomes possible to deblur inner defects. The mathematical description of the heat conduction equation serves to sharpen the initially blurred measurement results. As the LTC method transforms the thermal problem closer to a 1D model, it obviates the need for an inverse transform, enabling it to directly infer the internal structure from measurement results (excitation scenario with homogeneous response).

The LTC makes the measurement independent of the excitation frequency, and, given a sufficiently low excitation frequency and a sufficient number of iterations, it can detect the complete depth range at a high SNR up to the thermal penetration depth. Here, the features are displayed with a low size deviation. The BF influence disappears after a sufficient number of iterations, and the phase values become unique. A feature of the same size is displayed with decreasing phase difference and increasing depth. Thus, a feature’s depth can be established using the measured feature size and phase difference.

**Figure 3:**
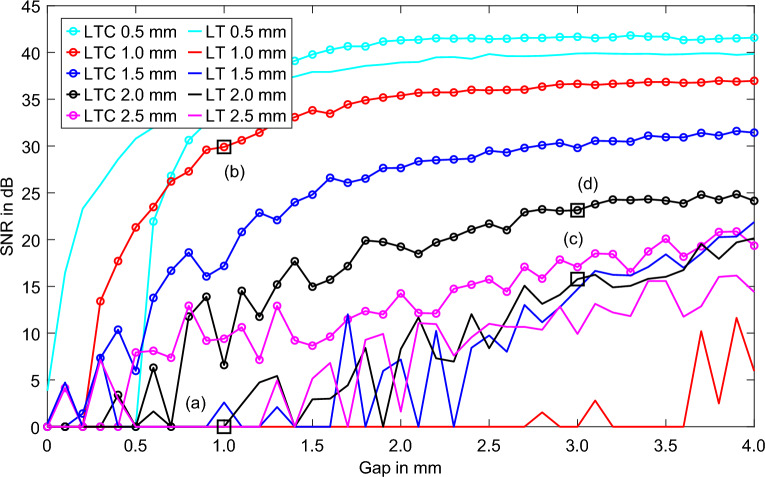
Feature separation of two squares with increasing gap in the depth range of 0.5–2.5 mm and at an excitation frequency of 10 mHz based on the SNR (dB) of the phase values.

**Figure 4:**
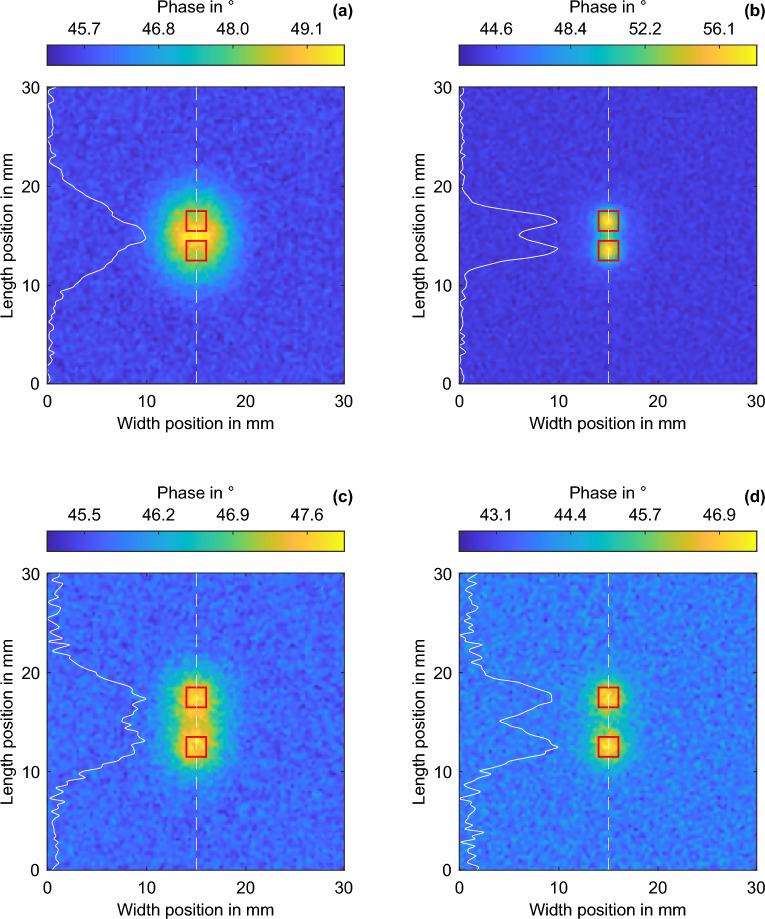
Simulated phase images of the (**a**) LT for a feature depth of 1 mm and a gap of 1 mm, (**b**) LTC for a feature depth of 1 mm and a gap of 1 mm, (**c**) LT for a feature depth of 2 mm and a gap of 3 mm, and (**d**) LTC for a feature depth of 2 mm and a gap of 3 mm. The red-dashed squares show the feature position. The white solid line describes the phase values scaled between the min and max at the dashed white line. The phase values of the LT are displayed physically negated.

To investigate the separability of closely spaced features, two square features of size 2 mm are compared at depths of 0.5–2.5 mm and gap distances of 0–4 mm from each other. The excitation frequency is 10 mHz, and the resulting thermal penetration depth is 2.16 mm. The LT phase range and the LTC range completely cover the range of investigation. Thus, LT can reliably detect large features in the investigation range. In all cases, the LTC is performed up to the tenth iteration. Figure 3 shows the separability of the two squares from a 0 mm gap up to a 4 mm gap based on the SNR. To determine the SNR, the simulation results of the vertical symmetry plane at a width of 15 mm are symmetrically averaged over a width of 5 px (total width = 0.5 mm), and the resulting line intersection is observed. In each case, the signal maximum is determined in the upper and lower halves of the line section (for LT and at a feature depth of 0.5 mm, the signal minimum is determined based on the change in the phase’s sign). If present, the signal minimum is recorded in between the two maxima (for LT and at a feature depth of 0.5 mm, the signal maximum is determined). The SNR is calculated from the minimum difference between the two outer values and the extremum that lies between them. The noise in the sound region of the simulation serves as the signal noise. Two depth–distance pairs with a depth and gap of 1 mm, as well as a depth of 2 mm and a gap of 3 mm, are selected from the studied space and labeled alphanumerically in Fig. 3. In Fig. 4, the simulated phase images of the LT and the LTC are compared. The phase values, represented by the white solid lines, are used for the separation of the two squares. The mutual interference of the features close to each other leads to a phase increase between the features and does not allow separation. Despite a spacing of 3 mm and a feature depth of 2 mm, the square features in (c) are also difficult to separate visually. However, in the separation by the extrema within the phase values, the features can be separated with a SNR of 15 dB. The corresponding phase images of the LTC are shown in (b) and (d). In both cases, the features can be clearly distinguished, and the feature shape is mapped precisely, despite the increase in noise. This is also reflected in the SNRs of separability: 24 dB for the 2 mm feature depth and 29 dB for 1 mm.

The BFD for the excitation frequency is approx. 0.8 mm (Fig. 2). Above the BFD, LT can detect features with a high SNR and low geometry deviation. Already, at the smallest feature distance of 0.1 mm, features can be separated at a depth of 0.5 mm in LT with a SNR of > 6 dB. The LTC exceeds the separability of the LT not until a gap of 1.1 mm. At the next feature depth (1 mm), the feature is only just above the BFD, and the geometric blur in LT is distinct. This is also shown in Fig. 4a. At an excitation frequency of 10 mHz and a depth of 1 mm, the features in LT can only be separated by a gap bigger than 3.7 mm. For LTC, however, the features in the 1 mm depth region can be separated with a SNR of > 6 dB starting from a gap of 0.3 mm. With increasing the feature depth, the separability of the LTC decreases slightly; however, in all cases, it allows the separation of the features with a SNR of > 6 dB, starting from a gap of 0.5 mm. For feature depths greater than 1 mm, the separability of LT increases; however, in all investigated feature depth regions, a gap of at least 2 mm is required to separate the features. LTC enables the imaging of features more sharply than LT, and closely spaced features can be separated. The resolution of closely spaced defects deeper than the BFD is improved through LTC by at least a factor of 4 compared with the results for LT.

**Figure 5:**
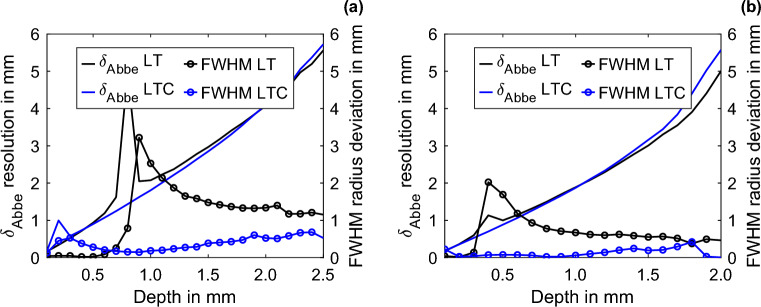
Comparison of the $\delta_{\rm {Abbe}}$ resolution and FWHM radius deviation over the depth for LT and LTC after ten iterations at excitation frequencies of (**a**) 10 mHz and (**b**) 25 mHz for FBH 2.

The ${\updelta }_{{{\text{Abbe}}}}$ resolution, as described in^18^ pertains to pulsed excitation at the depth of the defect and is determined by the highest angular frequency $\omega$ at which the signal, after traversing the depth *d*, remains just above the noise level. Here, the depth *d* represents twice the actual depth, as the thermal wave is initially excited on the surface, propagates to the backwall or to the FBH, and then returns to the surface. In the case of LT, there is only one excitation frequency, and the ${\updelta }_{{{\text{Abbe}}}}$ resolution limit is half the wavelength associated with this frequency (as illustrated in Fig. 4a, where this limit exceeds the 1 mm gap). However, with LTC, it becomes evident that this limitation can be surpassed, as illustrated, e.g., in Fig. 4b. Based on Eq. (1), the ${\updelta }_{{{\text{Abbe}}}}$ resolution according to^18^ for the measured SNR (Fig. 2d,f) is shown in Fig. 5 as a function of the feature depth at frequencies of (a) 10 mHz and (b) 25 mHz for the LT and LTC after ten iterations. The increasing feature depth and the associated decreasing SNRs cause the ${\updelta }_{{{\text{Abbe}}}}$ resolution to decrease. Except for the corresponding BFD, the curves of the ${\updelta }_{{{\text{Abbe}}}}$ resolution for LT and LTC match well and increase almost linearly with the feature depth. Above the thermal penetration depth, the LT provides increased SNRs and, thus (according to Eq. (1)), an improved lateral resolution. These values are compared with the radius deviations measured by the FWHM method for LT and LTC (cf. diameter deviations in Fig. 2g,i). Over the BFD, the FWHM radius deviation for LT is almost zero; it reaches its maximum at the BFD and subsequently gradually decreases. For LTC, the radius deviation increases only marginally as the feature depth increases. The FHWM radius deviation is small for LTC and largely independent of the feature depth. As shown earlier, if the LTC compensation is not yet complete, e.g., after ten iterations and at a frequency of 10 mHz up to a depth of ~ 0.6 mm, the LT shows a smaller radius deviation. Due to the small surface area of FBH 2, the feature can be approximately compared to the point source investigated in^18^.

Moreover, in^18^, the heat is directly generated at the feature and evaluated according to a Dirac delta heating, whereas for LT and LTC, the heat propagates from the surface to the feature and back and is evaluated trough the lock-in process. Even if a strict quantitative comparison between the two approaches is not valid, the geometry-preserving properties of LTC can be assessed by comparing the theoretical ${\updelta }_{{{\text{Abbe}}}}$ resolution with the geometry deviations measured by the FWHM method. The improvement of the lateral resolution by a factor of two after homogenization, as described in^20^, can be surpassed by LTC, compared with the results achieved with LT, over large depth ranges. Between the LT BFD and the thermal penetration depth, LTC can improve the FWHM radius-deviation accuracy by at least a factor of 2.2 at 10 mHz and 25 mHz. Averaged linearly over the depths, there is an average improvement of the FWHM radius-deviation accuracy at 10 mHz by a factor of 6.4 and at 25 mHz by a factor of 16.2 from LTC to LT. Both LT and LTC can overcome the resolution limits described in^18^. For LTC, the mean quotient between the ${\updelta }_{{{\text{Abbe}}}}$ resolution and the FWHM radius deviation is 8.6 at 10 mHz and 26.6 at 25 mHz.

### Description of the experimental validation

**Figure 6:**
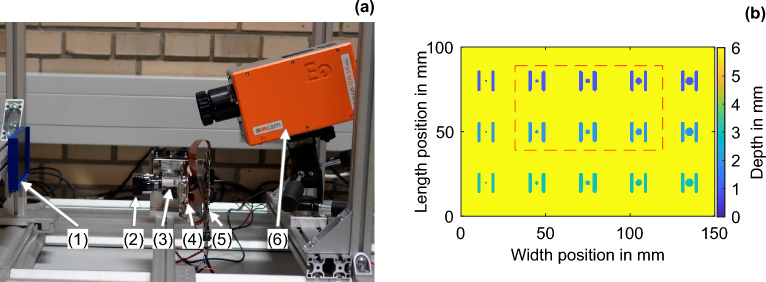
(**a**) Experimental setup for the LTC: (1) POM specimen, (2) 600 mm plano-convex lens, (3) ViALUX NIR CORE optics and CBM-90-IRD-X33-940 nm NIR LED, (4) Texas Instruments DLP650LNIR DMD chip, (5) ViALUX V-650L DLP® module, and (6) IR camera IRCAM EQUUS 327 k SM PRO. (**b**) Depth image of the POM specimen; the experimentally investigated measurement range is marked in red.

The experimental validation of LTC is performed using a NIR projector for excitation and an EQUUS 327 k SM PRO IR camera (IRCAM, Erlangen, Germany) for data acquisition. The NIR projector consists of a DLP650LNIR DMD chip (Texas Instruments®, Texas, United States), a CBM-90-IRD-X33-940 nm NIR LED (Luminus Devices, Inc., California, United States) with a 12 W light output power, and a NIR CORE optics component (ViALUX, Chemnitz, Germany). A 600 mm plano-convex lens is placed in front of the CORE optics to achieve the desired optical magnification. After losses due to the optics and the DMD chip, the NIR projector has a maximum light output power of ~ 7 W. At a working distance of 23 cm, the 1280 × 800 independent pixels of the NIR projector illuminate an area of ~ 110 mm × 69 mm, resulting in an *I*_max_ value of 920 W/m^2^. Additionally, the resolution results in an excitation pixel size of ~ 86 µm. The power emitted by the NIR projector and the pixel size are thus comparable to the excitation conditions applied in the numerical approach shown above. The resolution of the Camera is ~ 250 µm. As in the numerical part, measurements are made for two periods each. The compensation is performed offline, and the LTC takes ~ 30 min to complete six iterations at 25 mHz. If the projector control is directly linked to the thermography system, the inspection time could be reduced to approximately the product of the number of periods times the period duration times the number of iterations. At 25 mHz, this would be ~ 8 min until the sixth iteration. The experimental setup is shown in Fig. 6a. The technical feasibility of employing high-power NIR projectors for AT, based on the same DMD chip, has already been demonstrated in^29^. The micromirror array limits the incident beam power to a maximum of 160 W according to the manufacturer’s data sheet. A higher power than 7 W would further increase the performance or inspection area, as well as the SNR of the LTC. However, due to the LT measurement principle, the method already achieves good results even with low excitation intensities.

The experimentally investigated POM specimen has a dimension of 150 mm × 100 mm × 6 mm, and, according to the datasheet, its material properties are identical to those specified in the numerical part. Through laser flash measurements, however, the thermal conductivity was determined to be 0.46 W/(m K), which is subsequently considered for the comparative simulations below. This increases the thermal penetration depth by a factor of 1.22. Several pairs of grooves and intermediate FBHs are inserted into the POM specimen. A color-coded depth image of the specimen is shown in Fig. 6b. The grooves have length, width, and gap values of 12 mm, 2 mm, and 6 mm, respectively. The ligaments of the features are 1 mm, 2 mm, and 3 mm from the top to the bottom row. Symmetrically, between the grooves, the FBHs of the same ligament are inserted from left to right from 1 to 5 mm in diameter. A small part of the edge area of the projection surface is subject to increasing measurement artifacts with increasing iterations and is not considered in the evaluation. The measuring area investigated below is 87 mm × 49 mm and is marked in a red-dashed rectangle in the image of the specimen (Fig. 6b), as well as in the numerical comparison results (Fig. 8). In the four phase images, the horizontal intersections are indicated by dashed lines, and the corresponding phase profile is shown as a solid line of the same color. The phase profiles within an image are scaled to the maximum value of the phase profiles in the image. The white line intersections correspond to a feature depth of 1 mm, the cyan line intersections to a depth of 2 mm, and the yellow line intersections to a depth of 3 mm. The validation shows the comparison of the experimental LTC with the numerical LTC at exemplary excitation frequencies of 10 mHz and 25 mHz. Similarities between the simulation and experiments are observed at all excitation frequencies. The thermal penetration depths at excitation frequencies of 10 mHz and 25 mHz are ~ 2.63 mm and ~ 1.66 mm, respectively.

**Figure 7:**
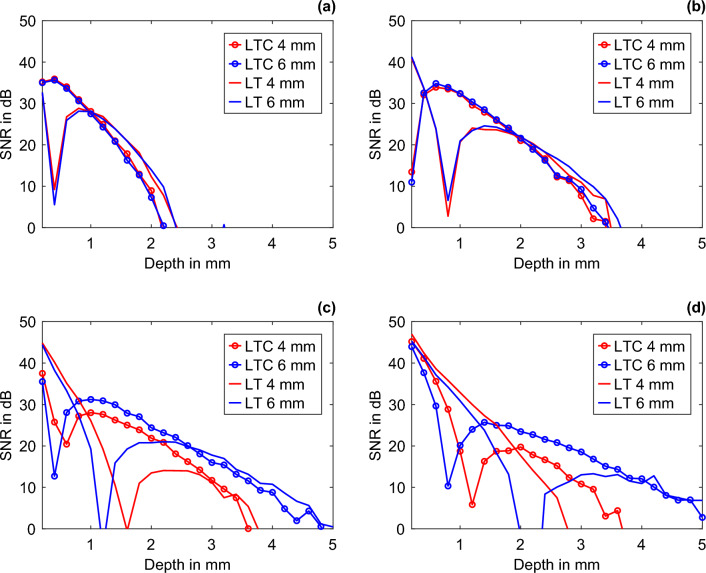
Numerical comparison of the SNRs of FBH 2 for different excitation frequencies and feature depths at plate thicknesses of 4 mm and 6 mm for the LT and LTC: (**a**) 25 mHz, (**b**) 10 mHz, (**c**) 5 mHz, and (**d**) 2.5 mHz.

The increase in the plate thickness from 4 mm in the simulation to 6 mm in the experiment exerts a minor effect on the measurement results at an excitation frequency of 10 mHz or higher. The plate thickness of 4 mm is ~ 1.53 times the thermal penetration depth and close to the inspection range of LTC. The backwall geometry, thus, only marginally influences the measurement results. At lower excitation frequencies, conversely, the range of the method increases so that the backwall geometry influences the measurement results (Fig. 7). For different excitation frequencies, the plots show the simulated SNR of the detectability of FBH 2 for LT and the tenth iteration of LTC for 4 mm and 6 mm thick POM specimens. The plots correspond to a vertical cross-section of Fig. 2d,f at a single excitation frequency and a thermal conductivity of 0.46 W/(m K). At (a) 25 mHz, the SNRs do not differ among the varied plate thicknesses. At (b) 10 mHz, the first marginal deviations can be observed for the LT at near-backwall features. At (c) 5 mHz and (d) 2.5 mHz, the backwall of the specimen strongly influences the measurement results, resulting in a clear difference between the SNRs of FHB 2 in the 4 mm and 6 mm thick POM specimens. With increasing the distance between the backwall and the feature, the detection increases at sufficiently low excitation frequencies. Furthermore, the LTC range for thermally thick specimens is always below the range of the corresponding LT measurement. The thinner the plate relative to the thermal penetration depth, the more the BFD shifts to deeper layers. Thus, as the frequency decreases or the plate thickness increases, the BFD shifts to greater depths. There is an exception (d), where the LTC range is larger than the LT range. Due to the BFD near the backwall geometry, the LT cannot detect a contrast between the backwall and the feature at great depths, but the LTC can. For hard-to-detect features near the backwall, LTC can provide significant improvement at low excitation frequencies, with the thermal penetration depths being in the same range as the plate thickness.

**Figure 8:**
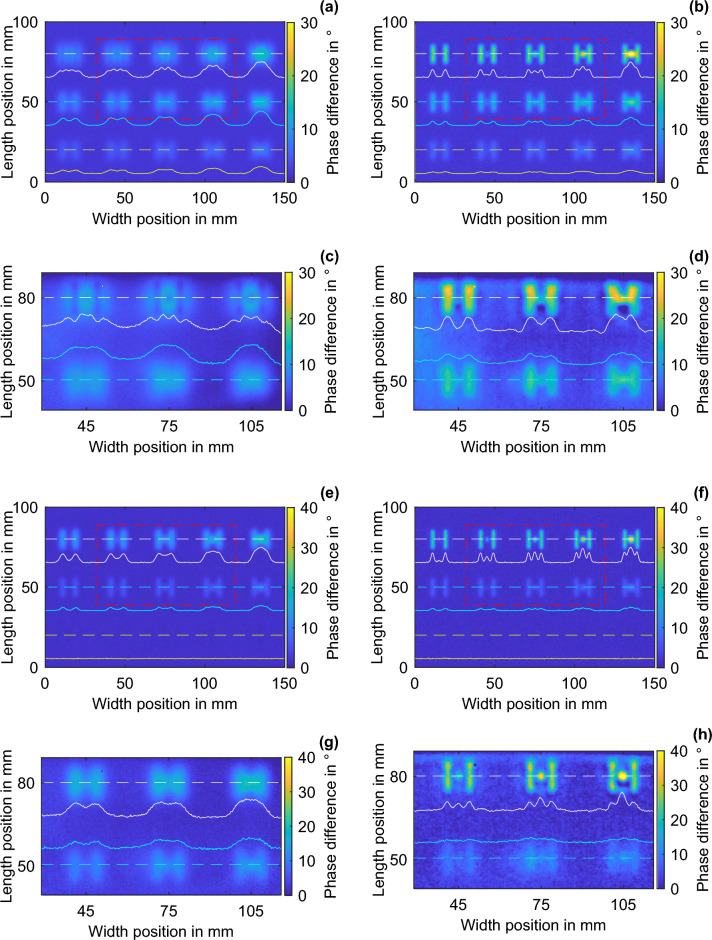
Comparison of the numerical and experimental LT and LTC results for a POM specimen with grooves and FBH. (**a**) Numerical LT at 10 mHz, (**b**) numerical LTC at 10 mHz, (**c**) experimental LT at 10 mHz, (**d**) experimental LTC at 10 mHz, (**e**) numerical LT at 25 mHz, (**f**) numerical LTC at 25 mHz, (**g**) experimental LT at 25 mHz, and (**h**) experimental LTC at 25 mHz. The LT phase differences are displayed physically negated.

As described in the method section, the amplitude and the offset decrease with the number of LTC iterations, which inevitably decreases the SNR. In the experiment, six iterations proved to be optimal. Figure 8a,b (10 mHz) as well as (e) and (f) (25 mHz) show the numerical results of the LT measurement and the sixth LTC iteration for the POM specimen shown in Fig. 6b. All feature agglomerates can be detected in the LT phase image at 10 mHz (Fig. 8a). However, the grooves and FBH cannot be imaged as independent features. The feature depth of the upper row is beyond the BFD; thus, the grooves and FBH only show a slight phase contrast relative to the sound background. Due to the local phase shift caused by the lateral heat flux, a contrast relative to the local environment is revealed. The same can be observed in the experimental validation (c). The SNR decreases with increasing feature depth. This agrees with the findings from Fig. 2d,g. In comparison, the LTC increases the SNR as well as the edge sharpness. The grooves and the FBH, starting from FBH 2, can be mapped at feature depths of 1 mm and 2 mm as individual features. Experimentally, these findings can also be confirmed for the feature depth of 2 mm. The LTC transforms blurred phase images of LT into geometry-preserving images and increases the SNR. Features beyond the thermal penetration depth (*µ*) are not shown as sharp features near the surface, and features beyond the range limit are not detected. The first is true for features at a depth of 2 mm (1.2 times the thermal penetration depth) and an excitation frequency of 25 mHz, and the latter is true for features at 3 mm. However, features below *µ* (here at 1 mm depth) are mapped by LTC numerically and are experimentally crisp with a high phase contrast (Fig. 8h). All the LT and LTC phenomena obtained numerically here could be confirmed experimentally.

## Conclusion

The major application area of LT is in the field of materials testing for validating the integrity of technical systems. This is essential for both the production and maintenance of safety-relevant components in transportation (planes, trains, ships, etc.), machinery (shafts, structures, etc.), etc. LT is also used in civil engineering (structure inspections) and cultural heritage sites to test materials (paintings, statues, etc.). We wish to emphasize that LT can also be utilized in biomedical applications. Several photo-acoustic and photothermal approaches for inspecting biological samples with remarkable spatial resolution exist. Independently of testing animate or inanimate matter, the amount of information gained from a measurement is essential. Here, the LTC is investigated numerically and experimentally, and its limitations are demonstrated. Compared with LT, LTC delivers improved defect detection and separation efficiencies, as well as improved defect spatial resolution. LTC reduces the dependence of the inspection results on the excitation frequency and the BF. Regardless of their depth, defects can be detected sharply up to the thermal penetration depth (*µ*). LTC is robust and independent of the specimen and can also compensate for unknown materials or inclusions. Defects of higher thermal conductivity (e.g., metallic foreign particles in plastics) can also be detected with this method. The phase difference is unique and decreases monotonically with the defect size and depth. The results show that LTC can improve the average FWHM radius-deviation accuracy of LT by factors of over 6 and 16 at excitation frequencies of 10 mHz and 25 mHz, respectively, at depths between the BFD and the thermal penetration depth. LTC overcomes the previous resolution limitations of AT without requiring time-consuming reconstruction algorithms. Furthermore, the resolution of closely spaced defects deeper than the BFD is improved through LTC by at least a factor of 4 compared to the case with LT. The amount of information in entropy terms is indicated by the SNR and spatial resolution, and our results show that both parameters can be significantly improved if non-adapted frequencies are used in conventional LT. This improvement in the spatial resolution will, in turn, increase the amount of information gained from the measurement. However, the application of LTC is limited by the temporal and spatial flexibility of the excitation source and the number of iterations. More powerful excitation sources are expected to increase the measurement area significantly. An IR Camera with improved resolution could increase the lateral resolution further. Additionally, a regressive iteration approach instead of the linear approach used in this work could compensate for the large phase and amplitude differences in the first iterations and, thus, reduce the number of required iterations and the measurement time. The goal is to further improve the LTC using modified compensation algorithms and to extend it to an online measurement so that only a few periods are required to achieve full compensation. As previously detailed, various approaches have been employed to deblur internal defects in AT. Unlike the described methods, LTC introduces significant alterations to the thermal wave field, resulting in a considerable reduction of lateral heat flux. This transformation aligns the thermal diffusion problem with a 1D model—a remarkable achievement made possible by LTC. The method relies on a robust iterative process that can be executed through pixel-wise mapping of the excitation and detection sources, even without knowledge of thermal properties. A prerequisite for the successful application of LTC is the initial detection of defects by LT, typically in a blurred state. Once these conditions are met, LTC operates autonomously without the need for additional input, surpassing both LT and most pixel-wise thermography evaluation techniques in terms of resolution and SNR. While the current excitation power appears to meet the requirements for future applications in the medical field, the nondestructive testing of larger-scale technical components, such as aircraft parts, requires a considerably higher excitation power level. The prospective widespread technical utilization of LTC hinges upon the advancement of NIR DLP-modules operating in the low-kilowatt range, as opposed to the mere tens of watts.

## Data Availability

The data used to support the findings of this study are available from the corresponding author upon request.
